# Using natural experimental studies to guide public health action: turning the evidence-based medicine paradigm on its head

**DOI:** 10.1136/jech-2019-213085

**Published:** 2019-11-19

**Authors:** David Ogilvie, Jean Adams, Adrian Bauman, Edward W. Gregg, Jenna Panter, Karen R. Siegel, Nicholas J. Wareham, Martin White

**Affiliations:** 1 MRC Epidemiology Unit and Centre for Diet and Activity Research (CEDAR), University of Cambridge, Cambridge, UK; 2 Charles Perkins Centre and Prevention Research Collaboration, University of Sydney, Sydney, New South Wales, Australia; 3 School of Public Health, Imperial College, London, UK; 4 National Center for Chronic Disease Prevention and Health Promotion, Centers for Disease Control and Prevention, Atlanta, Georgia, USA

**Keywords:** evaluation, natural experimental studies, non-randomised studies, practice-based evidence, public health policy

## Abstract

Despite smaller effect sizes, interventions delivered at population level to prevent non-communicable diseases generally have greater reach, impact and equity than those delivered to high-risk groups. Nevertheless, how to shift population behaviour patterns in this way remains one of the greatest uncertainties for research and policy. Evidence about behaviour change interventions that are easier to evaluate tends to overshadow that for population-wide and system-wide approaches that generate and sustain healthier behaviours. Population health interventions are often implemented as natural experiments, which makes their evaluation more complex and unpredictable than a typical randomised controlled trial (RCT). We discuss the growing importance of evaluating natural experiments and their distinctive contribution to the evidence for public health policy. We contrast the established evidence-based practice pathway, in which RCTs generate ‘definitive’ evidence for particular interventions, with a practice-based evidence pathway in which evaluation can help adjust the compass bearing of existing policy. We propose that intervention studies should focus on reducing critical uncertainties, that non-randomised study designs should be embraced rather than tolerated and that a more nuanced approach to appraising the utility of diverse types of evidence is required. The complex evidence needed to guide public health action is not necessarily the same as that which is needed to provide an unbiased effect size estimate. The practice-based evidence pathway is neither inferior nor merely the best available when all else fails. It is often the only way to generate meaningful evidence to address critical questions about investing in population health interventions.

## Introduction

Governments around the world are committed to tackling the growing burden of non-communicable diseases.^1^ Unhealthy patterns of behaviours such as smoking, diet, alcohol consumption and physical activity contribute substantially to disease risk and life expectancy, particularly in middle-income and high-income countries.^2^ The populations of lower-income countries are increasingly at risk of similar behaviour patterns as their economies develop. For example, insufficient physical activity is already estimated to account for nearly 10% of global premature mortality.^3^ The energy imbalance and metabolic health of people in lower-income countries are liable to worsen with the increasing availability of high-energy ultraprocessed foods and mechanisation of labour and transport that characterise an aspirational ‘Western’ lifestyle.

The aetiological associations between behaviours and many chronic disease outcomes are sufficiently well established to justify efforts to ameliorate behavioural risk factors. However, how population behaviour patterns might most effectively be shifted remains one of the greatest uncertainties for public health research and policy. To date, effort has largely been directed at developing and evaluating interventions to change the behaviours of individuals at higher risk, sometimes successfully. However, it is doubtful that merely scaling up these approaches to reach more and more people would be affordable, effective or equitable as a global disease prevention strategy.^2 4–6^ A more authentic and sustainable population-based strategy would complement the current focus on effective primary and secondary prevention—targeting individuals at higher risk—with more primordial prevention (table 1) that addresses the environments and policies that shape the circumstances in which we live. This vision has deep roots in the histories of public health and medicine.^7^ However, we remain largely ignorant about how to achieve it. This ignorance reflects the challenges in evaluating primordial prevention strategies of this kind, interpreting the findings and translating them into action. These challenges are reflected in the conclusions of recent systematic reviews, as exemplified in table 2.

**Table 1:** Glossary of selected terms

Term	Meaning in this paper
Decision-theoretical approach	‘A decision-theory approach utilizes relevant knowledge, theory and data both from (sic) observational and experimental studies to evaluate the likely efficacy of an intervention. If from this process it can be demonstrated that an intervention is sufficiently unlikely to cause net harm, then we can move to estimate cost-effectiveness. That is, we assess if the benefit relative to its cost is sufficient for the intervention to be recommended for application to population groups under consideration. This contrasts with the hypothesis-testing approach in which decisions about the efficacy of an intervention are made solely by using the findings of scientific studies that use statistical testing to evaluate their efficacy. The hypothesis-testing approach is central to evidence-based medicine but in practice groups charged with reaching decisions about health interventions for populations also use additional evidence alongside scientific, methodological and philosophical judgements.’^27^
Natural experiment	‘The term(…)lacks an exact definition, and many variants are found in the literature. The common thread in most definitions is that exposure to the event or intervention of interest has not been manipulated by the researcher.’^18^ ‘Natural experiments are, by definition, events that occur outside the control of the researcher. They are not ‘conducted’ or ‘designed’; on the contrary, they are discovered.’^22^
Primordial prevention	‘This term is advocated by some authors to describe elimination of risk factors(…)in contrast to primary prevention by reducing risks of exposure.’(S1)
Quasi-experiment	‘A situation in which the investigator lacks full control over the allocation and/or timing of intervention but nonetheless conducts the study as if it were an experiment, allocating subjects to groups. Inability to allocate subjects randomly is a common situation that may be best described as a quasi-experiment.’(S1)

Citation S1 in this table refers to the online supplementary reference list.

10.1136/jech-2019-213085.supp1 Supplementary data

**Table 2:** Key findings of recent examples of systematic reviews

Topic	Key points from main results and authors’ conclusions (emphases added)
Interventions to reduce ambient particulate matter air pollution and their effect on health	‘The evidence base, comprising non‐randomized studies only, was of **low or very low certainty**(…)Given the heterogeneity across interventions, outcomes, and methods, it was **difficult to derive overall conclusions**(…)The evidence base highlights the **challenges related to establishing a causal relationship** between specific air pollution interventions and outcomes(…)Researchers should strive to sufficiently account for confounding, assess the impact of methodological decisions(…)and improve the reporting of methods, and other aspects of the study, most importantly the description of the intervention and the context in which it is implemented.’(S2)
Fortification of staple foods with vitamin A for vitamin A deficiency	‘**We are uncertain** whether fortifying staple foods with vitamin A alone makes little or no difference for serum retinol concentration(…)**It is uncertain** whether vitamin A alone reduces the risk of subclinical vitamin A deficiency(…)The certainty of the evidence was mainly affected by **risk of bias, imprecision and inconsistency**.’(S3)
Nutritional interventions for preventing stunting in children living in urban slums in low‐ and middle‐income countries	‘Overall, the **evidence was complex** to report, with a wide range of outcome measures reported(…)**The certainty of evidence was very low to moderate**(…)All the nutritional interventions reviewed had the **potential** to decrease stunting(…)however, there was **no evidence of an effect** of the interventions included in this review(…)More evidence is needed of the effects of multi‐sectorial (sic) interventions(…)as well as the **effects of 'up‐stream' practices and policies**’(S4)
Environmental interventions to reduce the consumption of sugar‐sweetened beverages and their effects on health	‘**We judged most studies to be at high or unclear risk of bias** in at least one domain, and most studies used non‐randomised designs(…)**Implementation should be accompanied by high‐quality evaluations using appropriate study designs, with a particular focus on the long‐term effects** of approaches suitable for large‐scale implementation.’(S5)
Fortification of wheat and maize flour with folic acid for population health outcomes	‘**Most studies had unclear risk of bias**(…)Limitations of this review were the small number of studies and participants, **limitations in study design, and low‐certainty of evidence due to how included studies were designed and reported**.’(S6)

Source: The five most recently published systematic reviews listed on the website of the Cochrane Public Health Group (http://ph.cochrane.org/cph-reviews-and-topics, accessed 4 July 2019). Citations in this table refer to the online supplementary reference list.

In this paper, we discuss the growing importance of evaluating natural experiments in primordial prevention and their distinctive contribution to generating evidence for public health policy. We identify some of the obstacles to this type of research and suggest greater effort and investment in this area to ensure that research more effectively supports public health action.

## What do we need to know?

We should reflect on the extent to which our research is aligned with the societal processes it is intended to inform.^8^ To that end, we must distinguish between ‘behaviour change’ as a population *goal* on the one hand, that is, the *outcome* we ultimately wish to achieve; and on the other, ‘behaviour change’ as an intervention strategy or moral imperative, that is, a way of framing both the problem (people are making poor behavioural choices) and the solution (they need to make different choices). An approach too narrowly focused on people and their behaviour or lifestyle as the problem, and thereby on interventions that often seek ‘to persuade the poor to change their behaviour’,^9^ is not compatible with a social ecological understanding of the causes of ill health that are not amenable to individual control.^2 10^

The evidence available to guide policy has long been subject to an ‘evaluative bias’ in favour of behavioural interventions targeting people at higher risk because such interventions are generally easier to evaluate and, in particular, easier to randomise.^11^ This type of evidence tends to overshadow that for strategies that act on whole populations by targeting critical leverage points in the *systems* that generate and sustain less healthy behaviour patterns.^2 12^ This implies a need to direct greater policy and research attention to where the underlying problems are located: not merely among individuals at higher risk, nor even among groups of more deprived individuals, but in the more fundamental causes of ‘dis-ease’ (sic)^13^ in communities—for example, in the unhealthy environments created as a consequence of the structural conditions of the planning, transport and welfare systems and the housing and labour markets.^10^

## Why do we not know?

The lack of evidence for effective primordial prevention strategies may be traced to one of three types of obstacle.

The first is a set of *political* obstacles encountered by researchers who are willing but unable to produce the evidence. Researchers seeking to evaluate environmental or policy interventions—such as improving access to green space or taxing particular foods—depend on governments or other agencies to implement evaluable strategies.^6^ Because these interventions often entail greater political cost or risk than those focused on individual choice, they tend to be introduced less often. When such policies do find favour, demonstration projects and similar initiatives are often introduced quickly, without time to establish rigorous evaluation studies.^14^ Even if an intervention is both promising and evaluable in principle,^15^ an agile evaluative response may depend on more rapid and flexible sources of funding than have traditionally been available.

The second is a set of *cultural* obstacles in research, manifested by a research community that is able in principle to produce the evidence, but rendered somewhat unwilling by circumstances. Nearly two decades ago, it was pointed out that only a small fraction of UK public health research expenditure was directed towards ‘solutions’.^16^ Today, observational epidemiology and the development and evaluation of targeted behaviour change interventions remain easier and more secure routes to ‘doing something’, achieving funding, producing publications and career progression.^12 17^ A research community that, quite understandably, ‘follows the money’ in this way may therefore be distorting the agenda in research (and, consequently, in policy).

The third is a set of *practical* obstacles. Primordial preventive strategies are generally implemented as ‘natural’ or ‘quasi-’ experiments rather than ‘true’ experiments (table 1).^18^ Evaluating these strategies thereby makes for a more complex and unpredictable undertaking than, for example, a typical clinical trial—which is not without enormous potential challenges of its own. This calls for a particularly flexible and nuanced approach to natural experimental study design and analysis, along with sufficient capability and capacity to deliver this.^6^ No wonder, then, that it seems more common to see papers calling for this type of research than to see papers reporting it.

## Two complementary modes of evidence generation

**Figure 1:**
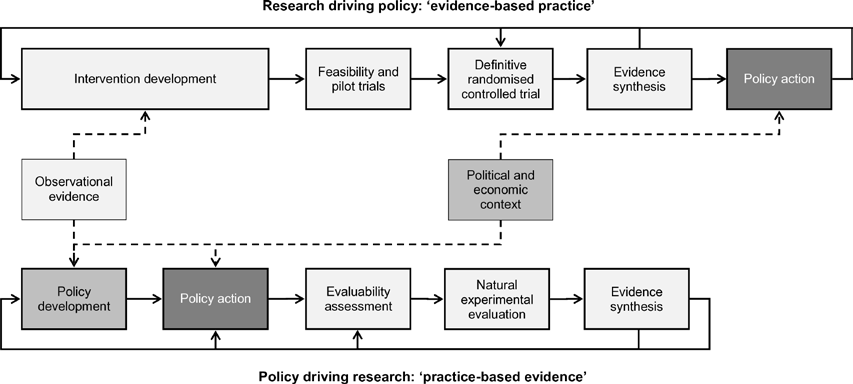
Two complementary modes of evidence generation.

Much has been written about the bench-to-bedside translational medicine pathway linking basic science with clinical practice. That concept has also strongly influenced thinking about evidence to support public health action, for example, in discourse that refers to institutionalising ‘proven’, ‘evidence-based’ interventions.^19^ It envisages a largely unidirectional pipeline in which researchers—informed by observational studies—develop interventions, subject them to feasibility and pilot testing, and then evaluate them in definitive randomised controlled trials (RCTs). These are often conducted in settings and groups of people unlike those in which a public health intervention might ultimately be applied. When a systematic review of multiple trials concludes that an intervention is effective, that intervention is regarded as ‘proven’ and may be recommended by a body such as the National Institute for Health and Care Excellence (NICE: www.nice.org.uk) for more widespread implementation, subject to broader contextual considerations such as affordability and political acceptability. This is shown in the upper pathway of figure 1. In this pathway, the purpose of evaluation can be seen as indicating whether a traffic light holding back an ‘unproven’ intervention should be turned from red to green.

However, this implied linear, rational way in which new knowledge is converted into ‘evidence-based practice’ has limited empirical support or practical utility for ‘upstream’ public health interventions.^19^ Even in comparatively well-resourced healthcare systems, major preventive initiatives such as cervical screening and routine health checks for over-40s have been introduced without evidence of effectiveness from RCTs to support them.^4 20^ This applies even more outside healthcare, where actions influencing complex systems of wider determinants of health such as food supply, income and urban planning are being taken all the time. These actions occur for a variety of reasons that may or may not be ostensibly concerned with health, and with or without evidence to support them or meaningful evaluation to learn from them.^2 6^ For example, when a new neighbourhood is built to accommodate unmet need for housing, planners make decisions about the mix of land uses, amenities provided (such as schools and parks) and street network layout. Each of these decisions constitutes an intervention that may influence physical activity and the risk of chronic disease among the people who live there.^21^ It is, however, unlikely to be realistic for governments to take no action until ‘sufficient’ evidence of all such effects have been cumulated and synthesised from multiple intervention studies.^22 23^

Public health research is concerned with ‘generating discoveries and new knowledge within the public health field itself’.^8^ This implies an opportunity and need to complement the evidence-based practice pathway, described above, with innovative solutions—generated in and for the real world by policymakers and practitioners—that can also be rigorously evaluated to produce ‘practice-based evidence’ (as shown in the lower pathway of figure 1).^5^ In this latter pathway, the purpose of evaluation can be seen as more akin to adjusting the compass bearing followed by existing policy rather than enabling a binary decision to proceed or not with the widespread implementation of a particular intervention. Whereas the former pathway depends on multiple instances of evidence of *effectiveness* to justify action, the latter depends more on multiple instances of *action* from which to develop at least preliminary evidence of effectiveness. This may in turn support taking further evaluable action and the consequent, cumulative reduction of uncertainty about its effects.

The application of each step of the practice-based evidence pathway, and how it differs from the converse pathway, can be illustrated with a worked example based on the published protocol for the ongoing evaluation of the UK soft drinks industry levy,^24^ a fiscal measure intended to reduce consumption (table 3). This particular intervention had not been implemented before, so the case for action was not based on established evidence of effectiveness as such. Rather, it rested on a plausible^25^ case for effectiveness based on a combination of observational epidemiology, simulation modelling and a limited set of evaluations of related interventions in other parts of the world. Without a political decision to ‘do something’ based on such ‘non-randomised’ evidence, it would be impossible ever to generate stronger evidence about effectiveness. Thoughtful assessment of the evaluability of the intervention revealed the complexity of its theorised mechanisms and potential outcomes, along with the unfeasibility of imposing an RCT design on this particular fiscal policy measure in this particular context. This dictated a natural experimental evaluation using a combination of *most appropriate* methods (eg, interrupted time series analysis) to systematically rule out alternative explanations for observed effects and demonstrate credible causal pathways leading to those effects.^25^ In contrast to the reliance on successful randomisation for causal attribution in an RCT, this study relies on integrating the findings of multiple quantitative and qualitative components for deriving robust inferences. These will contribute to subsequent evidence synthesis, as much in terms of validating (potentially generalisable) overall intervention theory as in terms of producing (context-specific) effect size estimates for meta-analysis.^6 26^ Such findings can be used to adjust existing policies, or inform future actions around the world, in this area to optimise their health outcomes.

**Table 3:** Natural experimental evaluation of the UK treasury soft drinks industry levy

Step in pathway	Précis of protocol (published in 2017)^24^
Observational evidence	Sugar-sweetened beverage (SSB) consumption is independently associated with multiple chronic disease outcomes. SSBs currently represent the single biggest source of dietary sugar for young people in the UK. Economic theory and data strongly suggest that price is an important determinant of SSB purchases.
Policy development	To reduce population consumption of SSBs, a range of interventions had been proposed, including fiscal measures. Globally a number of SSB taxes had been introduced, although few had been evaluated when this study was initiated. Modelling studies suggested important potential health gains, but no comprehensive evaluations had measured impacts on reformulation or consumption.
Policy action	In 2016, the Chancellor of the Exchequer announced a tiered soft drinks industry levy (SDIL) on industries importing or selling SSBs in the UK with the explicit intention of reducing consumption of sugar from SSBs. At the time of announcement, the UK SDIL was different from other SSB taxes: it was an industry levy (paid by manufacturers) rather than an excise tax (paid by consumers).
Evaluability assessment	The implementation of a fiscal policy is an intervention that is highly context dependent, resulting in reactions by many stakeholders including government, civil society, industry, health sector and consumers, and the potential to affect a range of diet and health outcomes. The SDIL was unique in its construction including a tiered levy directed at industry, and its 2-year lead time from date of announcement to implementation. Randomised controlled trials are recognised as the strongest method for determining causal effects. However, in the current context where the SDIL was introduced to the whole country at once, randomisation to intervention and control groups was not feasible.
Design of natural experimental evaluation	This evaluation seeks to improve our understanding of how such interventions evolve over time within complex food systems to influence products and purchasing, consumption and health outcomes. The evaluation will thus take a ‘systems’ perspective, aiming to evaluate a range of outcomes, associated processes and their dynamic interrelationships. Interrupted time series (ITS) methods offer one of the strongest quasi-experimental research designs. Using ITS designs, consideration of a range of outcomes (eg, SSB consumption declining as consumption of lower sugar alternatives increase) and mechanistic processes (eg, the relationship between price and purchases) can be explored such that a ‘pattern’ of impacts is appraised to provide the strongest possible basis on which to draw causal inference.
Evidence synthesis	Findings will be integrated and synthesised to develop a coherent overarching interpretation (and) test and refine the underlying intervention theory for the SDIL. Findings generated using different methods (qualitative, quantitative) could be triangulated to explore the extent to which they provide a consistent interpretation and conclusions about the impacts of the SDIL using pattern matching and causal process observation, thus strengthening causal inference.

## Towards a good enough evidence base for public health action

How, then, might effort and investment in developing the published evidence base more effectively support the kinds of policy intervention required for primordial prevention? Our analysis suggests three main implications; examples of potential actions arising from these are given in table 4.

**Table 4:** Examples of potential actions to help develop the evidence base

Arena	Potential action
Research training	The teaching of more established research methods in observational epidemiology, randomised controlled trials and meta-analysis in postgraduate training could be complemented with other mixed-method and natural experimental approaches more often taught and used in the social sciences
Research methodology	Efforts to develop consensus and guidance on the appraisal of natural experimental studies could be expanded, complementing the current emphasis on internal validity with greater consideration of external validity, transferability and utility for informing action
Research funding	Research funding bodies and their peer reviewers could assess natural experimental studies more closely on their own merits rather than using templates based too closely on the expectations of a typical randomised controlled trial
Academic publishing	Journals could adopt editorial policies committed to selecting manuscripts based more on the applicability of a given study design to a given research question than on prior assumptions about a hierarchy of study design
Policymaking	Policymakers could allow more time (and assign funding, if appropriate) to enable adequate theorisation, robust study design and baseline data collection to be undertaken before new policies and other interventions are implemented
Knowledge exchange	Policy and research communities could establish horizon-scanning or intelligence-sharing networks to bring implementers and potential evaluators into dialogue as early as possible in the process of establishing new interventions

### Intervention studies should focus on reducing critical uncertainties

Studies of interventions to change upstream determinants of disease risk, such as population dietary or commuting patterns, are sometimes criticised because they have not followed participants to ultimate, ‘hard’ physiological or clinical endpoints. However, there is no reason to expect that all parts of a putative causal chain should be directly proved within a single study. It has been argued that to inform action, public health intervention research should be guided as much by a decision-theoretical approach (table 1) as by the narrower, but more familiar, statistical hypothesis-testing approach.^27^ This implies that evaluation should focus on reducing the most critical uncertainties^28^ about what should be done—that is, the ways in which various intervention strategies influence population *behaviour* patterns—just as we judge smoking cessation services not on their direct impact on heart disease or lung cancer, but on whether they help people quit smoking.^29^ A case for (or against) action can be gradually cumulated using the iterative exchange of data and theory between empirical observational and intervention studies in a variety of contexts, simulation modelling of more distal or long-term health impacts and other sources of evidence.^30^

### Non-randomised study designs should be embraced rather than tolerated

Although natural experimental study designs have important theoretical underpinnings in common with the RCT, their worth does not reside solely in the extent to which they emulate an RCT design. Dunning proposes three criteria for assessing the utility of natural experimental studies.^31^ The first is that the allocation of an intervention can be treated ‘*as if*’ *it were random*, although not within a planned RCT. Although randomisation eliminates important sources of potential confounding, an expectation that intervention studies should entail a comparable allocation process (such as a lottery) would further entrench existing evaluative biases because many interventions relevant to public health are never likely to fulfil this criterion.^22^ This may be because randomisation is impractical (eg, new transport infrastructure is built in particular places for particular reasons) or politically unpalatable (if, eg, it is seen as withholding a service from certain areas or groups).^12^ Furthermore, intervention studies that ‘fail’ this criterion may pass with flying colours on Dunning’s other two criteria for utility. One of these relates to the *relevance* of the intervention to current, real-world policy questions. A key advantage of natural experimental studies is that they ‘do not interfere in the natural data generation process’,^32^ and thereby largely avoid the problems of ‘artificial and less directly informative’ inferences from effects observed in experimental studies in more controlled settings.^33^ The other criterion relates to the *plausibility* of the causal inference.^25^ Here again, a natural experimental study may be ‘more likely to generate causal evidence that applies to intervention implementation in real life’,^34^ particularly if it elicits evidence of *how* an intervention achieves its effects.^27^

Of course, this may appear to sit uneasily within a research funding system based on a biomedical paradigm that privileges the RCT above all other methods for establishing effectiveness.^35^ But randomisation does not necessarily hold the key to unlocking questions about public health action.^25^ Nor does the proliferation of epidemiological studies that link environmental exposures with health behaviours in a statistically robust way but are incapable of testing whether altering the former influences the latter.^21 36^ If a given method or study design is chosen for its alignment with the applied research question and executed in a rigorous and transparent way, it is likely to contribute important evidence even though (and perhaps *because*) it falls into the implicitly disparaging category of ‘non-randomised’ studies.^35^

### A more thoughtful approach to appraising the utility of evidence

This is not to deny that many non-randomised studies do have major limitations and are reported in ways that lack rigour or transparency. For example, systematic reviews of studies linking changes in the built environment with changes in diet, physical activity and adiposity have noted multiple potential sources of bias and that ‘studies with weaker designs were more likely to report associations in the positive direction’.^23 37^ In addition to all the issues that complicate the practice and interpretation of trials, in a natural experimental study close attention needs to be paid to understanding exactly what exposure to an intervention consists of; how an intervention comes to be assigned to some people, groups or areas and not others; finding a valid basis for estimating the counterfactual, such as by using a meaningful control group or a graded measure of intervention exposure; selecting and interpreting the adjustment for appropriate covariates to minimise the risk of confounding; and interpreting complex patterns within the outcomes, which may include divergent and potentially inequitable responses between subgroups, dose-response relationships and comparisons with multiple controls.^18 38 39^

We have well-established, and continually developing, catechisms for assessing the internal validity of intervention studies, and groups of studies, in health research.^40^ However, we lack clear consensus on the relative importance or interpretation of different aspects of internal validity in natural experimental studies, and therefore on how to make constructive use of an evidence base that fits poorly into existing appraisal systems.^6^ For example, current tools for assessing risk of bias appear predicated on a preference for studies that resemble an RCT as closely as possible.^22 23^ They tend to downplay or ignore the importance of ‘greater qualitative appraisal (and) theoretical and statistical knowledge’,^32^ and of what different quantitative and qualitative components of single or multiple studies might contribute in combination to a growing body of overall, more generalisable causal inference.^31 33^ In particular, we lack consensus on ‘how good is good enough’—which partly depends, of course, on the answer to the question ‘good enough for what?’ The complex evidence needed to guide public health action is not necessarily the same as that which is needed to provide an unbiased estimate of an effect size.

## Conclusion

We are more likely to halt the rise in the global prevalence of non-communicable diseases by taking and evaluating new, more ambitious or radical actions to address the underlying causes than by merely applying existing preventive approaches—even if these are effective—with greater intensity. Even apparently simple questions about effectiveness in this arena cannot be answered without action—although based on the best available evidence at the time—that necessarily precedes evaluation. The practice-based evidence pathway can be regarded as an essential, and currently under-resourced and undervalued, complement to the more established evidence-based practice pathway. It is neither inferior nor merely the best available when all else fails. On the contrary, it is often the only way to generate meaningful evidence to address critical questions about investing in population health interventions.

The two pathways for generating evidence described in this paper do not represent mutually exclusive approaches. Some policy and practice innovations could and should be evaluated in RCTs, and many more would benefit from more planned evaluation using a wider range of study designs. Nevertheless, the public health research community and those who fund and publish their work have key roles to play in supporting the development and credibility of researchers in this field, and the more thoughtful conduct, appraisal and synthesis of natural experimental studies, to populate critical missing pieces of the evidence base to support more effective public health action.

**What is already known on this subject**

- There are well-established associations between behaviour and chronic disease, which justify government efforts to reduce behavioural risk factors. However, the question of how population behaviour patterns might most effectively be shifted remains one of the greatest uncertainties for research and policy. This reflects the substantial challenges of evaluating population preventive strategies, interpreting the findings and translating them into action. Greater effort and investment in this area may help ensure that research more effectively supports public health action.

**What this study adds**

- We discuss the growing importance of evaluating natural experiments and their distinctive contribution to the evidence for public health policy. We contrast the established evidence-based practice pathway, in which randomised controlled trials generate ‘definitive’ evidence for particular interventions, with a practice-based evidence pathway in which evaluation can help adjust the compass bearing of existing policy. We propose that intervention studies should focus on reducing critical uncertainties, that non-randomised study designs should be embraced rather than tolerated and that a more nuanced approach to appraising the utility of diverse types of evidence is required.

## References

[1] General Assembly of the United Nations Political Declaration of the high-level meeting of the general assembly on the prevention and control of non-communicable diseases. New York: General Assembly of the United Nations, 2011.

[2] Marteau TM, White M, Rutter H, et al Increasing healthy life expectancy equitably in England by 5 years by 2035: could it be achieved? The Lancet 2019;393:2571–3. 10.1016/S0140-6736(19)31510-7 31258113

[3] Lee I-M, Shiroma EJ, Lobelo F, et al Effect of physical inactivity on major non-communicable diseases worldwide: an analysis of burden of disease and life expectancy. The Lancet 2012;380:219–29. 10.1016/S0140-6736(12)61031-9 PMC3645500 22818936

[4] Capewell S, McCartney M, Holland W NHS health checks – a naked emperor? J Public Health 2015;37:187–92. 10.1093/pubmed/fdv063 26022810

[5] Reis RS, Salvo D, Ogilvie D, et al Scaling up physical activity interventions worldwide: stepping up to larger and smarter approaches to get people moving. The Lancet 2016;388:1337–48. 10.1016/S0140-6736(16)30728-0 PMC5193005 27475273

[6] White M, Adams J Different scientific approaches are needed to generate stronger evidence for population health improvement. PLoS Med 2018;15:e1002639 10.1371/journal.pmed.1002639 30153252 PMC6112619

[7] Mackenbach JP Politics is nothing but medicine at a larger scale: reflections on public health's biggest idea. J Epidemiol Commun Health 2009;63:181–4. 10.1136/jech.2008.077032 19052033

[8] Viehbeck SM, Petticrew M, Cummins S Old myths, new myths: challenging myths in public health. Am J Public Health 2015;105:665–9. 10.2105/AJPH.2014.302433 25713962 PMC4358183

[9] Green J What kind of research does public health need? Crit Public Health 2014;24:249–52. 10.1080/09581596.2014.917813 10.1080/09581596.2014.917813

[10] Panter-Brick C, Eggerman M, Tomlinson M How might global health master deadly SINS and strive for greater virtues? Glob Health Action 2014;7:23411 10.3402/gha.v7.23411 24685169 PMC3970118

[11] Ogilvie D, Egan M, Hamilton V, et al Systematic reviews of health effects of social interventions: 2. best available evidence: how low should you go? J Epidemiol Community Health 2005;59:886–92. 10.1136/jech.2005.034199 16166365 PMC1732915

[12] Rutter H, Savona N, Glonti K, et al The need for a complex systems model of evidence for public health. The Lancet 2017;390:2602–4. 10.1016/S0140-6736(17)31267-9 28622953

[13] Krieger N Commentary: ways of asking and ways of living: reflections on the 50th anniversary of Morris' ever-useful uses of epidemiology. Int J Epidemiol 2007;36:1173–80. 10.1093/ije/dym228 18056125

[14] House of Commons Health Committee Health inequalities. third report of session 2008–09. London: Stationery Office, 2009.

[15] Ogilvie D, Cummins S, Petticrew M, et al Assessing the evaluability of complex public health interventions: five questions for researchers, funders, and policymakers. Milbank Q 2011;89:206–25. 10.1111/j.1468-0009.2011.00626.x 21676021 PMC3142337

[16] Millward L, Kelly M, Nutbeam D Public health intervention research: the evidence. London: Health Development Agency, 2001.

[17] Academy of Medical Sciences Improving the health of the public by 2040: optimising the research environment for a healthier, fairer future. London: Academy of Medical Sciences, 2016.

[18] Craig P, Cooper C, Gunnell D, et al Using natural experiments to evaluate population health interventions: new medical Research Council guidance. J Epidemiol Community Health 2012;66:1182–6. 10.1136/jech-2011-200375 22577181 PMC3796763

[19] Ogilvie D, Craig P, Griffin S, et al A translational framework for public health research. BMC Public Health 2009;9:116 10.1186/1471-2458-9-116 19400941 PMC2681470

[20] Cochrane AL, Holland WW Validation of screening procedures. Br Med Bull 1971;27:3–8. 10.1093/oxfordjournals.bmb.a070810 5100948

[21] Sallis JF, Cerin E, Conway TL, et al Physical activity in relation to urban environments in 14 cities worldwide: a cross-sectional study. The Lancet 2016;387:2207–17. 10.1016/S0140-6736(15)01284-2 PMC10833440 27045735

[22] Humphreys DK, Panter J, Ogilvie D Questioning the application of risk of bias tools in appraising evidence from natural experimental studies: critical reflections on Benton et al., IJBNPA 2016. Int J Behav Nutr Phys Act 2017;14 10.1186/s12966-017-0500-4 PMC5397808 28424086

[23] Benton JS, Anderson J, Hunter RF, et al The effect of changing the built environment on physical activity: a quantitative review of the risk of bias in natural experiments. Int J Behav Nutr Phys Act 2016;13 10.1186/s12966-016-0433-3 PMC5055702 27717360

[24] White M Evaluation of the health impacts of the UK Treasury Soft Drinks Industry Levy (SDIL) [protocol]. National Institute for Health Research: Southampton, 2017.

[25] Victora CG, Habicht J-P, Bryce J Evidence-Based public health: moving beyond randomized trials. Am J Public Health 2004;94:400–5. 10.2105/AJPH.94.3.400 14998803 PMC1448265

[26] Panter J, Guell C, Prins R, et al Physical activity and the environment: conceptual review and framework for intervention research. Int J Behav Nutr Phys Act 2017;14 10.1186/s12966-017-0610-z PMC5688667 29141646

[27] Threlfall AG, Meah S, Fischer AJ, et al The appraisal of public health interventions: the use of theory. J Public Health 2015;37:166–71. 10.1093/pubmed/fdu044 25015579

[28] Ling T Evaluating complex and unfolding interventions in real time. Evaluation 2012;18:79–91. 10.1177/1356389011429629

[29] Lancaster T, Stead LF, Cochrane Tobacco Addiction Group Individual behavioural counselling for smoking cessation. Cochrane Database Syst Rev 2017;101 10.1002/14651858.CD001292.pub3

[30] Diez Roux AV Complex systems thinking and current impasses in health disparities research. Am J Public Health 2011;101:1627–34. 10.2105/AJPH.2011.300149 21778505 PMC3154209

[31] Dunning T Natural experiments in the social sciences: a design-based approach. Cambridge: Cambridge University Press, 2012.

[32] Waddington H, Aloe AM, Becker BJ, et al Quasi-Experimental study designs series—paper 6: risk of bias assessment. J Clin Epidemiol 2017;89:43–52. 10.1016/j.jclinepi.2017.02.015 28351693

[33] Geng EH, Peiris D, Kruk ME Implementation science: relevance in the real world without sacrificing rigor. PLoS Med 2017;14:e1002288 10.1371/journal.pmed.1002288 28441435 PMC5404833

[34] Bärnighausen T, Tugwell P, Røttingen J-A, et al Quasi-Experimental study designs series—paper 4: uses and value. J Clin Epidemiol 2017;89:21–9. 10.1016/j.jclinepi.2017.03.012 28365303

[35] Petticrew M, Roberts H, Evidence RH Evidence, hierarchies, and typologies: horses for courses. J Epidemiol Community Health 2003;57:527–9. 10.1136/jech.57.7.527 12821702 PMC1732497

[36] Bauman AE, Reis RS, Sallis JF, et al Correlates of physical activity: why are some people physically active and others not? The Lancet 2012;380:258–71. 10.1016/S0140-6736(12)60735-1 22818938

[37] Mayne SL, Auchincloss AH, Michael YL Impact of policy and built environment changes on obesity-related outcomes: a systematic review of naturally occurring experiments. Obes Rev 2015;16:362–75. 10.1111/obr.12269 25753170 PMC4789114

[38] Trochim WMK Outcome pattern matching and program theory. Eval Program Plann 1989;12:355–66. 10.1016/0149-7189(89)90052-9

[39] Humphreys DK, Panter J, Sahlqvist S, et al Changing the environment to improve population health: a framework for considering exposure in natural experimental studies. J Epidemiol Community Health 2016;70:941–6. 10.1136/jech-2015-206381 27056683 PMC5390281

[40] Higgins J, Green S, eds Cochrane Handbook for Systematic Reviews of Interventions, Version 5_1_0: Cochrane Collaboration, 2011.

