# Supplementary material for: Using natural experimental studies to guide public health action: turning the evidence-based medicine paradigm on its head
Source: J Epidemiol Community Health. 2019 Nov 19;74(2):203–8. doi: 10.1136/jech-2019-213085 (PMC6993029; doi:10.1136/jech-2019-213085)
Supplement: Supplementary data [file jech-2019-213085supp001.pdf]

SUPPLEMENTARY REFERENCES

S1 Last J, ed. A dictionary of epidemiology, fourth edition. Oxford: Oxford University Press, 2001.

S2 Burns J, Boogaard H, Polus S, et al. Interventions to reduce ambient particulate matter air pollution and their effect on health. *Cochrane Database Syst Rev* 2019;5:CD010919.

S3 Hombali A, Solon J, Venkatesh B, et al. Fortification of staple foods with vitamin A for vitamin A deficiency. *Cochrane Database Syst Rev* 2019;5:CD010068.

S4 Goudet S, Bogin B, Madise N, et al. Nutritional interventions for preventing stunting in children (birth to 59 months) living in urban slums in low- and middle-income countries (LMIC). *Cochrane Database Syst Rev* 2019;6:CD011695.

S5 von Philipsborn P, Stratil J, Burns J, et al. Environmental interventions to reduce the consumption of sugar-sweetened beverages and their effects on health. *Cochrane Database Syst Rev* 2019;6:CD012292.

S6 Centeno Tablante E, Pachón H, Guetterman H, et al. Fortification of wheat and maize flour with folic acid for population health outcomes. *Cochrane Database Syst Rev* 2019;7:CD012150.
